# Harnessing the law to advance equitable cancer care in South Africa: exploring the feasibility, desirability and added value of a dedicated national cancer act

**DOI:** 10.3332/ecancer.2024.1658

**Published:** 2024-01-24

**Authors:** Salomé Meyer, Jane Harries, Julie Torode, Laurel Baldwin-Ragaven

**Affiliations:** 1 Cancer Alliance, Netcare Rehabilitation Hospital, 2 Bunting Road, Auckland Park, Johannesburg 2092, Gauteng, South Africa; 2 Cancer Association of South Africa (CANSA), 26 Concorde Road West, Bedfordview 2008, Gauteng, South Africa; 3 Global Health Unit, Institute of Cancer Policy, Kings College London. Strand, WC2R 2LS London, UK; 4 Department of Family Medicine and Primary Care, School of Clinical Medicine, Faculty of Health Sciences, University of the Witwatersrand, Parktown, Johannesburg 2193, Gauteng, South Africa; a https://orcid.org/0009-0006-5624-8170; b https://orcid.org/0000-0001-7359-8419; c https://orcid.org/0000-0002-9755-3968; d http://orcid.org/0000-0002-6744-3768

**Keywords:** legislation, South Africa, cancer care, cancer survivors, human rights, public health, cancer act, social responsibility

## Abstract

**Background:**

The 2017 World Health Assembly resolution on integrated cancer prevention and control provided clear guidance on creating an enabling environment for cancer care. Through a variety of mechanisms, including civil society advocacy, some countries have secured overarching legislation in the form of national cancer acts to promote equitable access and outcomes for cancer patients. In South Africa, cancer incidence is set to double by 2030; and, while existing legislative and policy frameworks do address cancer prevention and control, these are fragmented, poorly implemented and have had limited success.

**Methods:**

This study assessed the feasibility and potential impact of promulgating a dedicated national cancer act in South Africa through exploratory in-depth interviews with 25 purposively selected key informants from various stakeholder groups, including cancer survivors; legal scholars; human rights advocates; health care providers; public health specialists and academicians.

**Findings:**

Following thematic analysis, three key themes were identified: the content of a dedicated national cancer act, the socio-political leveragability of an act and accountability mechanisms that would support such an act.

**Conclusion:**

While most respondents had not considered the possibility of a dedicated national cancer act, they were open to the concept for South Africa. Concerns about widening inequities, duplication, funding and accountability would need to be addressed against the current backdrop of health inequities and limited human rights leveraging for health.

## Introduction

Cancer is a complex disease that requires intervention from all levels of the health care system (primary, secondary, tertiary and quaternary) and across the entire continuum of care, including access to highly specialised services provided by comprehensive cancer or referral centres as well as palliation [1]. In 2017, World Health Organisation (WHO) member states, including South Africa (SA), adopted World Health Assembly (WHA) Resolution WHA70.12 to guide national cancer prevention and control strategies in the context of an integrated approach [2]. Many countries have subsequently developed national cancer control plans (NCCPs) as overarching cancer strategies to steer state and non-state actors. NCCPs can be a means of securing state and non-governmental responsibility and accountability through plan approval, budget allocation, and ensuring the efficient use of allocated funds. Notwithstanding the intended aim of the NCCP ‘to reduce cancer incidence and mortality and improve quality of life of cancer patients through a […] plan that is systematic, equitable and evidence-based’ [3], recent analyses highlight challenges on the ground. NCCPs frequently do not address questions of equity in access to information, early detection and diagnosis [3–5]. Nor do they necessarily promote evidence-based standards of treatment, including palliative care [5, 6]. Further, in the African context, gaps in NCCP implementation remain, due to limited financing, inadequate service delivery, poor data collection and technical barriers [3, 7, 8]. In addition, while NCCPs guide policy and direct program development, they are not enforceable legal instruments that can advance human rights for cancer care [5]. Socioeconomic rights legislation as envisaged through the International Covenant of Economic, Social and Cultural Rights (1961) and the subsequent General Comment 13 on Article 12 (2000) sets out principles, standards, processes and procedures that are justiciable in countries that have ratified this treaty [9, 10]. In instances where human rights laws are not followed, there are consequences and remedies that define routes to prosecution and restitution [11]. The 2023 WHO publication *Health for All: Transforming economies to deliver what matters* highlights the importance of promulgating appropriately financed legal frameworks to ensure the attainment of the human right to health: ‘laws on paper need to be translated into laws in action’ through budgetary allocations [12].

Whereas national legislation affecting cancer prevention and control is mostly piecemeal, a number of countries over the years have passed dedicated acts to address cancer holistically and comprehensively. Four of these (Chile, Japan, Kenya and the Philippines) did so to augment their national cancer strategic frameworks (NCSFs), providing an integrated overarching legal scaffold for coordination and accountability. Table 1 summarises six acts highlighting their original focus as well as key lessons from that particular country’s experience. While these cancer acts span many decades and deal with different concerns, all have legally binding national coverage, extend the scope of cancer diagnosis and treatment and contain enforcement mechanisms. Notably absent in the older acts are human rights and equity considerations.

In the absence of dedicated national cancer acts, however, some countries have taken other significant legal steps to advance the rights of cancer patients. Australia as a high-income country passed the Cancer Australia Act 2006 that established a coordinating body for cancer control known as Cancer Australia as well as an advisory council [22]. In 2016, Uganda passed legislation in the form of a specific act to establish the Uganda Cancer Institute [23]. In Brazil, the National Cancer Institute (NCI) became linked to the country’s Unified Health System in 1980, to strengthen existing provisions, expand regulation and ensure more equitable access to cancer care [24]. In 2012, a further ‘*Law of 60 Days*’ was decreed to regulate the maximum patient wait time from diagnosis to initiation of cancer treatment [25]. As well, Mexico issued a ‘*Care Leave*’ decree in 2019 to allow insured parents to take partially paid leave from work up to 364 days to care for children under the age of 16 who have been diagnosed with cancer [26].

In South Africa, a country of roughly 60 million people, cancer incidence is set to double with a projection of 216,336 new cases by 2030 [27, 28]. While South Africa does not have a dedicated national act to address cancer comprehensively or adequate investment in coordinated prevention and control, there are numerous policies and legal frameworks relating to cancer. Table 2 lists the pieces of legislation that have elements governing cancer prevention and control, all of which derive from Section 27 and other guarantees within the Bill of Rights of the South African Constitution (1996) [29, 30]. In addition, it highlights two dedicated cancer policies for breast and cervical cancer.

While the South African Constitution entitles everyone to the right to access health care services, including reproductive health, the necessary accountability to respect, protect, promote and fulfil this obligation is missing, as is recourse on individual and systemic levels. In addition to health, Section 27 of the Bill of Rights provides for ‘food, water and social security’ and underscores ‘that the state must take reasonable legislative and other measures, within its available resources, to achieve the progressive realisation of each of these rights’ [31]. An analysis of such additional legislative measures reveals serious limitations as regards cancer prevention and control. The National Health Insurance (NHI) Act is hailed as the future to achieve health equity through universal health coverage (UHC) in South Africa [46]. However, there is no clarity on how cancer will be managed within the NHI, since benefit packages must still be determined [47]. Further, the National Public Health Institutes Act (NAPHISA) (Act 1 of 2020) has not yet been gazetted [42]. Even though this act makes provision on paper for disease coordination, cancer surveillance, training and research for ‘major health challenges’, it is unclear exactly how this act will achieve equitable cancer care. Broad health legislation, namely the National Health Act No 61 of 2003 [32], the Medical Schemes Act No 131 of 1998 [35] and the Medicines and Related Substances Act No 101 of 1965 [33], govern access but do not address specific diseases or services. Chapter 4 of the National Health Act No 61 of 2003 stipulates that the actual delivery of health care services is a delegated function to provincial authorities. Implementation of health policies is thus dependent on the financial and human resource capacities of each province, notwithstanding its receipt of an equitable share from the National Treasury according to a particular formula [32, 48]. Further, the limited powers of the National Department of Health (NDoH) to intervene in the provincial implementation of policies and guidelines add to the disintegration of cancer care services and health services in general.

Regarding the cancer specific policies mentioned in Table 2, the current structure of the NDoH does not allow for a concentrated cancer-specific focus, as cancer falls within the remit of more than one directorate across different divisions. For example, the prevention of liver cancer through neonatal hepatitis B vaccination and cervical cancer with the human papillomavirus (HPV) vaccine, is embedded within the Expanded Immunisation Programme [49] as well as housed in different directorates. Furthermore, there can be a lack of sustainability as regards programs, their evaluation and oversight. The Ministerial Advisory Committee on Prevention and Control for Cancer, which was appointed in 2013 [50] and provided direction from central government with a draft Breast Cancer Policy, did not have their mandate renewed. These national challenges reflect a piecemeal approach in that cancer prevention and control remain largely uncoordinated and fragmented, and subsumed within a myriad of other national health priorities.

Despite such elaborate legislative and policy frameworks, implementation of these provisions on the ground has remained a challenge. To be fair, these limitations were acknowledged in the most current National Cancer and Palliative Care Strategic Frameworks 2017–2022 [51, 52]. However, concrete measures to address the gaps in implementation have been slow to follow. It should be noted that South Africa’s strategic plans and frameworks developed to address cancer, such as the National Strategic Plan for the Prevention and Control for Non-Communicable Diseases 2022–2027 [53], the National Cancer Strategic Framework 2017–2022 and the National Policy Framework for Palliative Care 2017–2022 exist as national guidelines. Unlike legislation, these strategic mechanisms in themselves have no legal imperative to improve outcomes for patients by expanding cancer care [54]. The NCSF intends to strengthen cancer treatment facilities as one of the six goals to drive improvements; however, it lacks assigned responsibility and accountability mechanisms that would be necessary to operationalise activities countrywide, such as across rural-urban and public-private divides. Similarly, the other five goals that are to strengthen: governance and leadership on cancer prevention and control; human resources for cancer prevention and control at all levels of the healthcare system; technology, equipment, vaccines, medicines and infrastructure for cancer; surveillance and research on cancer; and, to explore and ensure sustainable financing for cancer prevention and control [51] did not have sufficient budget and implementation strategies to guarantee success. The National Palliative Care Strategic Framework has been extended until 2024, whilst the NCSF remains in effect until an update is available. This is to allow the alignment of these two strategic frameworks with the National Strategic Plan for the Prevention and Control of Non-Communicable Diseases (Meyer S, Personal communication with NDoH, October 2023).

With the official end of apartheid in South Africa in 1994, the newly elected democratic government faced what has been termed the ‘quadruple burden of disease’. This insurmountable ‘cocktail of four colliding epidemics’, consisting of poor maternal child health outcomes, infectious diseases such as HIV/AIDS and tuberculosis (TB), NCDs, violence and injury [55], placed enormous strain on a fragmented and incapacitated public administration that had to meet the health needs of the entire population rather than a select few [56]. Several decades later, the SA health care system remains one of the most inequitable in the world. Service delivery in the public versus private sectors and between provinces remains grossly unequal [57]. More than 80% of the population depends on public health services for cancer care, whereas 80% of the cancer health care professionals and treatment centres are based in the private sector [57]. Even though this sector has more than sufficient access to cancer care services, patients are often faced with out-of-pocket expenses for high-cost medicines that force them into poverty or accessing public sector treatment in an already overburdened public sector.

It is well documented that public sector patients frequently are diagnosed with late-stage diseases that are more challenging to treat, with high costs to the health system and little chance of cure, exacerbated by long waiting times for confirmatory diagnosis and initiation of treatment [58–64]. Treatment centres are largely attached to tertiary academic institutions with health sciences faculties that also have the responsibility of training oncology professionals.

It is against this backdrop of burgeoning cancer incidence, a stated commitment on the part of the government to redress the legacies of the past through human rights entitlements, and the increasing frustration of civil society and cancer advocacy groups at the slow pace of service delivery that we undertook to explore the feasibility, desirability and added value of a dedicated national cancer act in South Africa.

## Methods

### Study setting, recruitment and participants

This study took place in South Africa between August and November 2022. Sixty potential study participants were purposively identified by the research team to ensure broad inclusion of stakeholders and elicit a wide range of viewpoints. These key informants included cancer survivors who had previously accessed cancer care services in the public and private health sectors, cancer care service providers and health professionals, public health specialists, legal experts, human rights activists, those with pharmaceutical industry experience, health policy decision makers and academics.

After identification, the research coordinator (Salomé Meyer) sent an e-mail inviting each person to participate in this research study. Following an indication of their willingness and availability to be interviewed during the study period and an informed consent process, participants were recruited for in-depth interviews. From this outreach process, 27 respondents volunteered to participate. Two were unable to schedule interviews in the allocated time, resulting in a final total of 25 participants.

### Ethical considerations

Ethical approval to conduct the study was obtained from the Human Research Ethics Committee (Medical) at the University of the Witwatersrand, Johannesburg (Certificate M220473). Participation was voluntary and written informed consent was obtained prior to the interview, including consent to audio record the interviews. Confidentiality of study participants was provided by assigning each participant an identification number and anonymising responses in this manuscript. All quotations have a unique interview number and brief description of attribution to maintain anonymity.

### Study design and data collection

Individual in-depth interviews were conducted with each key informant. The research team collaboratively developed an interview guide to elicit participants’ views on the feasibility of a dedicated cancer act in South Africa, which explored the following areas.

1. Current limitations of cancer care in the public and private health sectors in South Africa.
2. Role of current health legislation and policies, including whether Section 27 of the SA Bill of Rights provides adequate protection for the right to health in relation to cancer care.
3. Knowledge of existing international cancer acts, and perceptions of their ability to leverage health equity and human rights-based cancer care.
4. Crucial requirements/content for any new dedicated cancer act in South Africa.
5. Appropriate accountability mechanisms for any future cancer act.

All interviews were facilitated by one of the authors (SM) who has 25 years of experience in cancer research and advocacy, and who was also the study coordinator for this project. Participants were offered and largely took advantage of receiving the interview guide and supplementary information in advance of the interview (i.e., basic cancer statistics and information on services as well as summary information on the cancer acts from the six countries, noted above). Interviews were conducted virtually in English using the Zoom videoconferencing platform. The duration of the interviews was between 45 and 90 minutes depending on the range and depth of views provided by the participants. All interviews were digitally recorded and then initially transcribed using Otter.Ai software, with subsequent corrections made manually after verification against the recordings.

### Data analysis

Data were analysed qualitatively using a thematic analysis approach [65]. The research team, consisting of all authors, reviewed the transcripts of the 25 interviews. It was agreed that data saturation in terms of topics and perspectives had been achieved. No additional respondents were invited to participate. Overarching themes were identified, with sub-themes extracted from within these. Agreement between the research team was sought through several virtual meetings and discussions. Cross-checks were also made to ensure that the analysis did not reflect biases, such as elite bias (e.g., being more influenced by the dominant voices or from those ‘better qualified’) or concertedness bias (e.g., selection of comments that seemed more specific).

## Results

### Participant characteristics

Of the 25 participants interviewed, ten were men and 15 were women. All participants were qualified in the specific stakeholder fields for which they were recruited. Six of the respondents were cancer survivors with extensive experience of cancer care in the public and private health sectors. Five respondents were legal experts, with a further five possessing robust knowledge of human rights and the application of human rights in health. Six of the interviewees were health care professionals closely involved in providing cancer care services in both public and private sectors, representing oncology, surgery, paediatrics, nursing and general medicine. Seven respondents were public health specialists. One respondent identified as representing the cancer pharmaceutical industry and a further two had backgrounds in pharmaceutical policy and access to medicines. Nine respondents stated that they were directly involved with health policy at either a national or provincial level or through academia.

The views of respondents varied depending on their specific expertise in the cancer care field and their convictions that a dedicated national cancer act would lead to change. There was consistency, however, across the spectrum of interviewees that there is a glaring disconnect between existing legal and policy frameworks and the protection of human rights as they relate to cancer care services in South Africa.

### Themes

Responses fell into three overarching themes: i) content suggestions for any dedicated national cancer act; ii) socio-political leveragability of such an act; and iii) accountability mechanisms to support an act. Additional sub-themes and patterns were also identified.

The quotes below represent the key overarching issues that emerged from the interviews and demonstrate the range of participants’ views.

### Content suggestions for a dedicated cancer act

#### Rationale for a dedicated national cancer act: why implement an act and what should it contain?

Defining comprehensive cancer care in accordance with the cancer continuum of care was important to set the stage for in-depth interviews. Whilst only five respondents had prior knowledge of dedicated cancer acts elsewhere in the world and had an opinion on their relative value, most respondents conceptually supported the idea that a dedicated cancer act would indeed make a difference in how cancer is managed from a human rights perspective and would in fact provide cancer patients with appropriate legal recourse. However, there were also concerns about how implementing such an act would be possible within the context of a poorly managed, fragmented and inequitable health care system.

An additional advantage according to a cancer administrative expert would be to establish a new central independent body that would standardise care and treatment protocols and act in a coordination role:

‘*If there is a National Cancer Institute, which is representative and transparent, and that is regulated by a cancer act … *[it could]* really coordinate the effort that it takes to manage cancer care across the whole continuum*’ (015).

The ability of a dedicated act to provide a comprehensive and integrated approach to the prevention and control of cancer on an equitable basis resonated with the majority of respondents as the main reason why they would support a dedicated act. One government official, however, expressed how on the one hand an act could leverage human rights to expand cancer care, while also noting a potential risk for lowering the quality of services:

‘*If there is an act, it must realise issues around equity and affordability and access, but it must not compromise care*’ (023).

A clinician treating cancer patients expressed concerns about further marginalising those without resources to exercise their rights under a new act suggesting that community mobilisation could ‘mitigate against inequity’:

‘*Another potential pitfall we need to watch out for is inequitable access. You enhance access by creating this legal framework. But not everyone has the health or legal literacy to be able to challenge the framework. So *[the Act]* could inadvertently advantage people who are in a position to do so versus those that don*'*t*’, while noting: ‘*but you do a lot of community organisation and mobilisation and education, you can mitigate against the inequality or inequity issue, too*’ (012).

#### Role of health activism

The role of health activism came into play as a reminder of what it takes to advance health rights. Given South Africa’s rich history of advocacy linked to expanding health care services, reference was made to the success of litigation in obtaining national antiretroviral therapy for people living with HIV/AIDS:

‘*You have to remember that the *[HIV] *prioritisation only came as a result of activism*’ (024).

A human rights lawyer added another view on why a dedicated cancer act would be significant, as cancer is unique in terms of treatment underscored by a growing cancer ‘pandemic’:

‘*So, they apply *[law]* just as they would to other diseases, which is a shame, because cancer is not like other diseases, and it cannot be treated like other diseases. And, you know, the fact that we don*'*t have a cancer act as yet is just another indication that the response to cancer, as a growing essentially pandemic in South Africa, is not being addressed*’ (025).

#### Funding an act

Concerns about funding a dedicated act were raised as a potential stumbling block, together with both the necessity and possible pitfalls of a dedicated focus on cancer juxtaposed against other conditions such as NCDs and mental health. A health systems expert explained:

‘*Cancer is a special disease that needs specialists, special environment, special equipment, special human resources as well as the infrastructure. It*'*s a challenge*’ (021)*. *

Another respondent reflected that a dedicated cancer act might open up one disease for too much scrutiny within the context of limited resources:

‘*It will enhance cancer specifically*. . . [with possible]* unforeseen ramifications…* [It might cause] *deviation of *[health]* resources from other programmes … because something is being measured, looked at and can be prosecuted, you could inadvertently shift resources from *[other important diseases] *that are not being focused on … widening existing inequities*’ (012).

A human rights activist noted that legislation without a dedicated budget or fiscal allocation would make implementation challenging, as it would have no sway:

‘*It needs to have a budget, and it*'*s been well established in our law for a long time. A policy without a budget is not a legal policy, because you can*'*t implement a policy without a budget*’ (024).

This was further supported by a public health expert in government who felt that it would be more appropriate to have a dedicated funding stream rather than an act:

‘*In a way, it might be more appropriate to ask for the funding than to ask for the act, because funding can be more easily linked to a quantified plan with gaps, whereas an act doesn*'*t necessarily do that*’ (009)*. *

#### Political contestations

Another concern about having a dedicated cancer act was raised by a human rights legal expert with activist experience, in light of potential political interference. The current delegation of powers to provincial authorities for the delivery of health care services as prescribed in the National Health Act was identified as a possible barrier to the successful implementation of a dedicated act. This person noted that provincial authorities have to provide budget and resources for effective service provision, which is influenced by the political party governing a particular province and its alignment with the national ruling party.

‘*One of the arguments you can make is that often you have *[the] *example of the Western Cape cancer programme being better or different to Limpopo *[Province]*. That inconsistency is a violation of non-discrimination, equality, dignity … If you want an act, and you*'*re saying it*'*s under national law and a national competency, then there has to be some norms and standards, basic norms and standards which each province has to then put into effect*’ (004)*. *

Further highlighting the political divide between those in government and a population in need, one cancer survivor explained:

‘*The biggest conflict that we*'*ve got in South Africa is the conflict between the interests of the party and the interests of the people. That*'*s the fundamental conflict. And … no legislation is going to fix that*’ (001).

#### Implementation issues

One health care professional and activist pointed out that South Africa already has excellent public health policies and oversight mechanisms; however, it is the actual implementation of these measures that is lacking coupled with little recognition of the existing constitutional guarantees around access to health care. Adding anything more would be problematic:

‘*We have so much policy that is unimplemented that I have great reservations about putting *[in] *anything else, when they don*'*t even listen to the Constitution. So, we have, from a policy perspective, almost a failed state*’ (003).

A pharmaceutical industry and public health expert remarked on the inability of political leaders to implement relevant regulations in support of the country’s overarching National Health Act:

‘*The National Health Act allows the Minister *[of Health]* to make regulations on the selection of essential medicines. He*'*s chosen not to issue those regulations. And they*'*ve not been issued now for *19* years. Irrespective of who the incumbent is, he*'*s also chosen not to implement a lot of other regulations*’ (010).

One cancer survivor raised the issue of when to sequence the implementation of a cancer act:

‘*I*'*m in two minds about *[an act]*. We definitely need it. But I don*'*t see how having it before we actually have access and suitable treatment is going to help. So, I think we need to actually go and jack up our healthcare system as it is, then we can put something in a legal framework*’ (002).

Speaking to a health care system in crisis and a dysfunctional state, a seasoned health and human rights activist viewed the introduction of a new cancer act as potentially futile, and requiring much more effort at this point in time:

‘*The whole *[health]* system is in an Intensive Care Unit *[in crisis]*. COVID has obviously aggravated that. An edit to that is that we have a pretty dysfunctional Parliament. To try proposing a cancer act … that*'*s going to take you 15 years. … When you*'*ve got the executive, legislature and judiciary in different states of crisis, it*'*s so much harder*’ (004)*. *

### Socio-political leveragability of a cancer act

#### Addressing inequities and limitations in cancer care in South Africa

Respondents uniformly highlighted the failure of the current health care system to provide adequate cancer care in South Africa and thought that an act might assist in raising awareness and potentially eliminate inequities. Care received in both public and private sectors was viewed by respondents as problematic for different reasons, including the concentration of services in urban areas, the lack of patient centred care and empathy. A cancer survivor and public health expert explained:

‘*The private health sector is not comprehensive and not patient-centred … There are times when there*'*s over investigation, overtreatment, also times when there*'*s under investigation and under treatment, so both are a problem The distribution of the private services is also problematic from an equity point of view … it*'*s the usual concentration in urban centres*’ (005)*. *

Rural provinces have limited cancer care services, increasing the burden of the disease, pain and suffering. A cancer survivor and public health and administration specialist described this limitation as: ‘*If you happen to be elsewhere *[in provinces without comprehensive cancer care services]* you may just be missed*’ (019).

Lack of resources in rural provinces contributes to the overall poor experience of cancer services. This was supported by the views of a health systems expert in cancer who commented on the lack of empathy and support from health care professionals working in rural settings:

‘*The nurses…they*'*re burnt out because they*'*re operating in an environment that is not conducive. They are operating with ayikho *[nothing]…*it is depressing to operate in that environment where you are giving quality care. You don*'*t see that rigour that used to be there *[in nurses]*, you will just see the gogos *[grandmothers or older women]* who are just dragging themselves. Sometimes even when listening to them when addressing these patients, there is no love*’ (021).

In discussing the inequities within the district health system, where access to particular medication formularies depends on the level of care – primary to quaternary – to which patients present, a health systems expert in cancer reflected that:

‘***…****if the medicines are only distributed according to the size of a health facility, then it*’*s a problem. Medicines should not be distributed according to the level of the hospital. It*'*s not fair that some taxpayers cannot get the medicine *[they need]* even if he/she is in a primary care facility, compared to a patient who is in a tertiary care who can*’ (021)*. *

A pharmaceutical expert shared their view on the central need for fairness:

‘*Key principles of fairness, which is equity of access to care whether you are geographically compromised, or you are economically compromised, you should still have fair access to care*’ (022)*. *

#### Voices of cancer survivors

The voices of cancer survivors were possibly the strongest in terms of supporting the fact that current policies do not provide enough protection or promote equity. Respondents confirmed that the existing NCSF and dedicated cancer policies for cervical and breast cancer are not enough to provide a human rights-based legal framework justiciable for everyone. A cancer survivor with a legal background supported the view that stronger legal measures are required to advance equity in the health care system:

‘*Despite the fact that you have all these policies and legislation in place it doesn*'*t guarantee your health care, cancer care. And that*'*s what we are looking for. We want it legislated. And we want to guarantee that if you are diagnosed with cancer, this is the process … when you got the cancer, you*'*re taking care of yourself, but you*'*re fighting the system as well to get the treatment. So, you almost want to create a system where if you*'*re diagnosed, and it*'*s not necessarily only for cancer, but for anything, but we are speaking about cancer, specifically, you are diagnosed, the system actually takes care of you. And you focus on your healing*’ (006)*. *

#### Opportunities and limitations within existing legal frameworks to leverage health rights

Given that there is no national cancer act in South Africa, respondents reflected on how the existing legal protections for access to health care services have been leveraged, successfully or not. A human rights and legal expert expressed strong views when referring to examples where constitutionally guaranteed health rights in the Bill of Rights had been tested as to their justiciability, citing cases that eventually reached the Constitutional Court but with decisions that fell short of expectations:

‘*What really failed the people of South Africa is the interpretation of that right *[Section 27 to access health care services]*. The Constitutional Court, in its powers in applying the Constitution, has a lot of reach. It can interpret the current Bill of Rights in the Constitution, to extend the protections given to the people in South Africa to include rights to health*’ (025)*. *

In particular, the Constitutional Court judgement in the 1997 Soobramoney v. Minister of Health (Kwazulu-Natal) matter, which contested government-imposed restrictions on haemodialysis for renal failure, reinforced the acceptability of progressive realisation of health care rights over time as well as the autonomy of provinces to regulate health service delivery, making it nearly impossible for any patient to take legal action against the state. According to the same respondent, applications of the interpretations of the Constitution have been inadequate:

‘*The Constitution gives the *[Constitutional] *Court the power to interpret the rights and the policy decisions made by government to say that in the instance where there is a need, or there*’*s a dire need, for particular socio-economic rights to be addressed, the government must reprioritise to make sure that that need is addressed. And I don*'*t think that came out in that *[Soobramoney] *judgement. And since then, it*’*s been an uphill battle to try and get the protections under that section. So, you know, the writing in a constitution might be what it is, but the interpretations have been wholly inadequate, and so have the applications of those interpretations*’ (025)*. *

Furthermore, a cancer survivor felt that people are not well informed about their health rights and how to attain them. For individuals, accessing legal remedies remains an impossible task. Only by working through dedicated human rights focused organisations would one be able to achieve any success:

‘*The only way you can use it *[the law]* is to go to the courts in terms of Section *27*…. That*'*s all you can do. Even if you get that judgement, whether it*'*s actually implemented is another story. To enforce the judgement is not the easiest. We know that. Which then becomes a question of how easy it is to access legal services …If you work with organisations, like Section *27 [a health rights NGO]*, or the public interest law sector, they can do it. But if you go through the private route, it will cost you a pretty penny ... So that*'*s another story about access. You would probably die quicker than getting the judgement*’ (006)*. *

A legal expert with health and human rights experience did note successful precedent in leveraging the Constitution with another disease process, and therefore considered its potential applicability to address cancer concerns:

‘*Thinking of the judgement in the TB case in prisons, where the Court said that *[the Department of] *Correctional Services must ensure that treatment gets to people in correctional facilities... I do think that Section *27 [of the Bill of Rights]* can definitely be used, in a similar vein, for accessing cancer care*’ (014).

#### Do we need a new act, or can we better leverage the legal protections we already have?

Others highlighted how adjustments to existing legislation might be more helpful than creating a new act. One legal expert pointed to insufficient workplace protections for cancer patients as well as discriminatory practices that could be addressed directly through bolstering the Labour Act, or even extending the existing protections for people living with HIV to those living with cancer to prevent dismissal as a result of their diagnosis and treatment:

‘*If they*'*re just small amendments… [trade] unions can go to their members and say we got to have this benefit *[for cancer patients too]*, and that would be a more pragmatic way of making some gains on the employment side*’ (018)*. *

The proposed NHI Bill to introduce UHC in South Africa is regarded as the mechanism that will create more equity and homogeneity between private and public health care sectors. A cancer survivor and legal expert indicated that a dedicated act would further provide a platform for implementing NHI principles in cancer care:

‘*If you have a public healthcare system, despite the fact that I*'*ve got pre-existing conditions, I*'*ll have access to it, because the legal framework would apply that, and it would free up funds for me to use elsewhere*’ (006).

Measured against the existing National Health Act and proposed NHI Bill, the added value of a dedicated cancer act was questioned by a pharmaceutical policy expert:

‘*I*'*m not sure if it would make that much difference. The issue of equity is broader than cancer. It goes right across all diseases, and ensuring an equitable way of treating is what was intended with the National Health Act. I think it missed the boat, in terms of the extent to which it included and covered the private sector. The governance structures are not there. They don*'*t talk to issues of access, they talk to issues of confidentiality and informed consent for treatment, but they don*'*t talk to access issues. Access is really something that at the moment is only dealt with in the Medical Schemes Act and is intended to be dealt with under the NHI*’ (010).

### Accountability mechanisms inherent in an act

#### Current health accountability frameworks

Even though accountability and oversight mechanisms are built into existing legislation that give rise to such entities as the Parliamentary Portfolio Committee for Health, these structures are not used appropriately. A public health expert remarked:

‘*The oversight role of Parliament is something that*'*s very poorly developed and poorly executed. We need more public hearings in Parliament, where Parliament has to show what it*'*s doing with that data, what its oversight is, and opens up to civil society and patient organisations to interrogate*’ (010).

Moreover, the delegation of powers to provincial departments adds to the lack of accountability according to a cancer survivor and public health cancer expert:

‘*The present accountability links to the provincial Department of Health, *[whereas]* the NDoH *[National Department of Health]* has very little accountability for coordinated cancer care at the present time. … if there was a coordinated strategic central planning committee that was representative that they would also have to be accountable for the ultimate quality of the services*’ (011)*. *

In relation to the establishment of core standards for health care service delivery, one public health expert and academic indicated that compliance is not currently benchmarked against human rights targets:

‘*Whatever act provisions are made, it has to look at meeting standards of care that are compatible with the Constitution*’ (013)*. *

The role of the Auditor General (AG) in relation to accountability and recourse regarding the current public health situation in the country, together with the Public Protector’s Office, was raised by a legal health expert with a health and human rights background:

‘*There should be an auditing element to this, where the AG can come in and audit to see are things working the way that they should be working. And if not, the AG is able to blow the whistle to say, things are not working the way that they should be working. Legislation gives them teeth, but maybe they haven*'*t been using the teeth as much as they can. As much as they are empowered to, of course, they don*'*t have the same teeth that the Public Protector has, who has a much stronger if I could put it that way, a much stronger teeth, in the sense that they are able to. Their reports are binding and have to be implemented*’ (014)*. *

#### Imagining accountability in a new act

The ability of a dedicated act to hold government accountable for public sector health care was expressed by several respondents. One experienced health activist explained:

‘*When there*'*s an act, *[it’s]* something that you can hold government to account on*’ (007).

Another respondent, a pharmaceutical policy expert, saw a national cancer act as a potential accountability mechanism for the private sector as well:

‘*It *[a dedicated cancer act] *will make governments and medical schemes* [private health insurers]* much more accountable; it will be embedded in human rights principles*’ (008).

As one cancer survivor stated, embedding accountability into an act would mean that a clear entity becomes responsible for ensuring service delivery for cancer patients:

‘[Currently], *we have no accountability. If you did put this into legislation, then you could at least identify who*'*s responsible. And that*'*s a good first step towards accountability*’ (001).

## Discussion

This research explored the feasibility, desirability and added value of establishing a dedicated national cancer act in South Africa through interviews with a range of 25 key informants from government, civil society, human rights organisations, patients, cancer health care providers, the legal profession and academics. Broad themes emerged from this study, which included the content, socio-political leveragability and accountability mechanisms of such an act.

Most study participants were unaware of other countries’ initiatives in promulgating national cancer acts, and had not considered this as a possibility for South Africa. However, once explained, they were open to and curious about the idea, often citing the need for civil society mobilisation to define and support such an act. Interestingly, this reflects our unpublished findings on the passage of national cancer legislation in Japan, the Philippines and Chile, which harnessed cancer advocacy from a unified cancer community, largely taking a bottom-up approach.

Study participants also provided important insights and experiences of existing SA health law, human rights and the accountability mechanisms that are required to achieve equitable cancer care. They highlighted disparities in the diagnosis and treatment of cancer patients in the public sector, pointing out significant divides between rural and urban settings, while also speaking to limitations and gaps in the private sector. Cancer survivors, in particular, spoke from a place of intimate knowledge about the challenges accessing the requisite care during their illnesses.

### Content of a national cancer act in South Africa

Emerging from a spirit of redress, participants thought that an act would enforce equitable access to cancer care between different levels of care, between sectors and across the cancer continuum of care, with suitable patient protections. Some spoke of the need to establish a well-resourced national coordinating body for cancer prevention and control to direct national policy and ensure deliverables. This body, for example, might define a comprehensive basket of cancer services, extend treatment availability and reduce inequities.

Although not probed specifically, the strategic priorities to expand government funding for cancer research, and to train more oncology and palliative care practitioners were not identified by the study participants as possible content in an act. The 2020 SA 10-year strategy to achieve a sufficient workforce for UHC lays out the abysmal rates of medical specialists in the country [66]. Whereas there were 16.5 specialists per 100,000 population in 2019, these were maldistributed between the public and private sectors: with ‘seven specialists per 100,000 population employed in the public sector and 69 per 100,000 in the private sector’ [67]. The specialty of radiation oncology was even more skew, with 15 times more of these specialists working in private practice settings [67].

### Leveragability of a national cancer act

In theory, leveraging a singular national act for cancer seemed appealing to participants, especially in light of inequitable access to treatment and the provincialisation of health service delivery that causes fragmentation and variations in standards of care. Aware of the growing cancer burden and frustrated by the lack of implementation of previous and current cancer policies, participants were able to envisage how a dedicated act might assist reduction in cancer incidence and mortality. South Africa already has two acts dealing with specific health conditions, standardising approaches and treatment. The Mental Health Care Act 17 of 2002, as amended [68], repealed obsolete laws that governed mental health care and ushered in a ‘human rights orientation with the intention to ensure humane care with appropriate accountability’ [69]. Yet, even with this Act, resources for appropriate care remain inadequate, and there is bureaucratic indifference to lobbying from health professionals and mental health users to amend and update the Act [69]. The Choice on Termination of Pregnancy (CTOP) Act 92 of 1996 as amended, similarly repealed apartheid-era legislation that severely restricted abortion and affirmed new constitutional protections of ‘the right of persons to make decisions concerning reproduction and to security in and control over their bodies’ [70]. Hailed initially as a victory for abortion rights, and augmented by concerted efforts from the NDoH to implement the CTOP Act [71], severe limitations in expanding access persist, with the demand far exceeding the health system’s ability to provide adequate, timely, safe and respectful services [72–75].

Some participants imagined that a national cancer act would be a way of obtaining much-needed financial resources for cancer, compelling National Treasury to create a dedicated funding stream. In South Africa, financing is often viewed as a marker for commitment or political will. Following wins in the courts, HIV has received dedicated programmatic funding, including at the provincial level, for more than a decade. This includes funding of the SA National AIDS Council for R28.9 million (almost $1.6 million USD) in 2023/2024 and four HIV NGOs to the tune of R163.9 million ($8.6 million USD) per annum. In contrast, government funding for all NCDs together with associated NGO support was a combined total of R3 million (approximately $160,000 USD) per year for the period 2018–2023, pointing to serious inaction from the government to respond to the global call to focus on NCDs in an integrated manner [76]. These figures contrast with estimates that the increased cancer burden in the SA population will cost more than R50 billion (approximately $2.6 billion USD) by 2030 [27, 56, 77], creating a sense of desperation that perhaps an act would force government to provide the necessary resources.

### Accountability

Almost all participants bemoaned the lack of government accountability for the non-delivery of mandated health care services generally, and the non-implementation of cancer policies specifically. Ideally, a national cancer act would set out clear goals with milestones, delegate responsibilities and create reporting mechanisms as well as establish consequences for either wilful neglect or negligence.

Some participants were of the view that since an act would accord enforceable rights, it would be a powerful tool to take a national framework for cancer prevention and control to the next level. Implementation failures of previous and current cancer policies without any consequences indicated, for some, that policy alone will struggle for impact. Furthermore, policies are often dictated by the agenda of the political party in power, with no continuity following a change in government. In contrast, legislative provisions are not easily amenable to such political fluctuations. As relates to cancer prevention and control, an act would institute a standard against which to assess government actions, with the added value of justiciability, to be used as necessary to litigate against impunity.

### Ambivalence about a dedicated act

Whether a dedicated cancer act is indeed the route to adopt in South Africa is not clear as participants highlighted various challenges. Participants pointed to instances of failed policy implementation by state actors, flagrant disregard of court orders and an inability to execute constitutional imperatives effectively. They also questioned the readiness of the dysfunctional health care system to tackle cancer prevention and control at this juncture. Finally, the amount of time and energy it would take to draft a comprehensive national cancer act and shepherd it through the different legislative processes versus the pay-off, was tactically uncertain. Given the urgency to intervene to address the burgeoning cancer burden, many participants appealed to pragmatism and shorter-term solutions. Suggestions included harnessing existing legal mechanisms, such as the country’s labour laws, as well as piggybacking on to the recently acceded NHI Bill.

Given that the NHI bill ‘seeks to provide for universal access to health care services in the country in accordance with the NHI White Paper and the Constitution of South Africa’ [78] and commits to address issues of equity, quality, efficiency and affordability, it too can be harnessed to address specific cancer-related challenges [43]. Since many of the technical aspects of NHI implementation have to be determined, opportunities remain to intervene and enable equalisation and improved funding for cancer care. Some participants believed that linking cancer care with this NHI process offers more potential rather than initiating a separate stand-alone cancer act and legislative process, which might take more than a decade.

Many respondents were well-informed about historic efforts in South Africa to extend health rights through litigation, citing both positive and negative examples of such cases. On the positive side, ‘[t]he judicial enforcement of socio-economic rights has in fact demonstrated a potential to balance oscillations in legislations and policies that sometimes inflict adverse consequences on the poor as a result of defective political decision making. […T]hrough political intransigence, the state has in some instances not complied adequately with its socio-economic rights obligations, which has allowed the courts the latitude to require the state to justify its actions or in-action’ [79]. Although access to anti-retroviral treatment to prevent mother-to-child transmission of HIV was extended by the Constitutional Court to pregnant women in 2001 [80, 81], other attempts to expand health care services through legal challenges have not been as successful, as in the Soobramoney v. Minister of Health (KZN). Furthermore, South Africa has been criticised for extensions of socioeconomic rights adjudicated by the legal system as responses to litigation, rather than proactively expanded through parliamentary legislation or policy mechanisms. This has led to charges of judicial overreach as well as controversy over the separation of state powers [11, 79].

Finally, concern around the slow, exclusionary and expensive process of legal proceedings was another reason that participants expressed hesitation in adding new human rights legislation. There were insights into the parallels about how ordinary individuals who do not have access to health care services would similarly not gain access to the courts to further litigate their rights.

Ultimately, it must be decided whether a dedicated national cancer act in South Africa can really address the gaps in the cancer continuum of care. Study participants highlighted the enormous challenges that the state already has in meeting its current obligations put forward by existing health legislation and policies. There are concerns that another act will lie fallow as well, inadequately utilised for its intended purpose.

### Reflections on our study findings

It is indicative that regardless of the professional title, illness experience or other positionality, mass dissatisfaction was expressed about how the state and the private sector are each dealing with cancer prevention and control in South Africa. While perhaps not fluent in the details of current legal, policy and strategic frameworks, participants uniformly highlighted gaps related to not only how these mechanisms were structured in the first instance but also the failures of implementation. Such recognition and insight provide fertile ground for additional exploration and engagement on how things might improve, especially with expressions of intent to increase civil society activism, lobby government and speak to power across the health system. In this respect, lessons from other countries listed in Table 1 above may prove instructive. Kenya as a regional best practice example used their strategic framework to transition to a dedicated Cancer Act in 2012 [82]. Japan moved from a 10-year strategy for cancer control established in 1989 to a dedicated Cancer Act in 2006 with active civil society involvement [83–85]. The Philippines moved away from a NCCP to a dedicated cancer act with strong civil society advocacy [86].

In South Africa, PHC will be the initial focus of the proposed NHI. If an integrated cancer benefits package would be included in NHI, this could go a long way towards increasing equitable access across the public and private divide [87]. Yet, even the Brazilian NHI system, hailed as one of the most comprehensive in the world, had to ensure additional protections for cancer care through dedicated legal decrees [88]. In South Africa, expanding cancer services will place additional demand on health care professionals for early detection and screening. Without dedicated and funded cancer policies and guidelines, and adequate training of health care professionals and community health care workers, this might even accentuate risk given the collapse of private sector resources [89–92]. Providing mass screening and detection in the absence of effective referral pathways and adequately resourced treatment facilities downstream can further exacerbate challenges of timely treatment.

However, these are not reasons to avoid prioritising cancer prevention and control within NHI. Evidence from other countries points to the fact that bolstering cancer care services while expanding UHC potentially contributes to general health systems strengthening and broad economic and health gains in the long run [93–95]. Investment in human capital and equipment for radiological imaging can further add value to illness detection and treatment, for example in the fields of cardiovascular disease and nutrition [96–98]. We remain hopeful that the challenges highlighted in this study can be addressed through concerted action to maximise cancer provisions within the NHI, to establish a national cancer coordinating mechanism to bring together the current fragmented piecemeal approach, such as a NCI, and, finally, to continue to explore the feasibility, desirability and added value of a unified national cancer act.

Lastly, although not mentioned by our study participants, it is understood that a national cancer act would never replace existing legislation and policies highlighted in Table 2, nor the SA NCCP. As in other countries, the hope would be that an overarching national cancer act would serve to augment these and other efforts through improved coordination, financing and legal enforcement.

### Strengths and limitations

This is the first and only study to elicit the experiences and viewpoints of a select group of key stakeholders about legislative remedies to improve cancer care in South Africa. It provides a preliminary understanding of the potentials as well as pitfalls for a dedicated national cancer act. Deeper engagements with legal scholars on the practicability of a national cancer act in the SA context as well as distilling the lessons from other countries, especially low and middle income countries (LMICs) with similar budgetary constraints, would be necessary to pursue. Finally, specific engagements around the role of new therapies and the framing of value-based care on equitable access must happen urgently as the country’s NHI plans advance. Further exploration of local or smaller scale remedies, perhaps through provincial engagements, could also be useful.

## Conclusion

While most study participants had not considered the possibility of a dedicated national cancer act, they were open to the concept for South Africa to achieve equitable population-based cancer care that is aligned with the WHA Resolution 70.12 and other WHO guidance. Concerns about widening inequities, fragmentation, funding and implementation would need to be addressed against the current backdrop of government dysfunctionality and historically constrained human rights leveraging for health.

## List of abbreviations

NCCPs: National cancer control plans; NCSF: national cancer strategic frameworks; NI: National Health Insurance; NCDs: non-communicable diseases; NDoH: National Department of Health; SA: South African; UHC: universal health coverage; WHA: World Health Assembly; WHO: World Health Organization.

## Conflicts of interest

The authors declare that they have no conflicts of interest.

## Funding

Funding for this study was received from the 2021 MSD Independent Oncology Policy Grant Program. The funder was not involved in any aspect of the research or write up of the findings.

## Author contributions

SM, JT, JH and LBR:

- Contributed significantly to the conception and design of the research project.
- Participated in data analysis, including the development of themes.
- Drafted, critically reviewed and edited the manuscript for important intellectual content.
- Approved the final version of the submitted manuscript.
- Attest to the accuracy and integrity of the work and agree to be accountable for any questions or concerns.

SM was solely responsible for data collection and wrote the first draft of the manuscript.

## Figures and Tables

**Table 1.** Six countries with dedicated national cancer acts and their intentions.

Country	Year enacted	Focus, stated intent and limitations
United Kingdom (UK) [13]	1939	Initially passed to protect patients from quackery in cancer therapies, this Act subsequently provided for National Health System payment for cancer prevention, diagnosis and treatment. Human rights language is not included explicitly.
United States of America (US) [14]	1971	Given the high burden of disease, this Act focussed on research, prevention, treatment and cures of for cancer. It established the NCI with dedicated public research funding, of which Cancer Moonshot [15] is an extension. It was passed in the context of a declared ‘War on Cancer’. Health rights are not included in US Constitution and do not feature here.
Japan [16]	2008	Japan has a high burden of disease. With civil society advocacy and political commitment, it moved from NCSF to a dedicated act. Health is recognised as a human right in the Constitution.
Kenya [17]	2013	Kenya is the first LMIC to develop a national cancer act, which conforms to international norms and standards. The Act is linked with the NCSF and coordinating mechanism (a NCI). It supports the right to health in the Constitution, together with a NHI program. Civil society and patient advocacy organisations were involved in its passage.
Philippines [18]	2019	Provides the most extensive scope of all existing cancer acts in terms of defining beneficiaries, protection of vulnerable groups, devolution of responsibilities and overall approach to cancer care. The Act is linked with disability legislation and budgetary provisions, as a result of civil society advocacy. Oversight is established by the National Integrated Cancer Control Council. The right to health is enshrined in the Constitution.
Chile [19]	2020	Decrees were issued to support this cancer act, which established the National Cancer Commission, following civil society advocacy due to a high disease burden. Although ‘the right to the protection of health’ was enshrined in Chile’s first Constitution (1925), and retained in subsequent versions, the current constitution effectuated under the Pinochet regime in 1980 is ‘tainted by authoritarianism from its origin, and promotes a subsidiary role of the state in health’ [20]. Although a National Health Fund is in place, inequities persist [21].
LMIC: low and middle income country		

**Table 2.** Selected South African legal and policy frameworks that support cancer prevention and control.

	Year enacted	Provisions
**Legal mechanisms**		
Bill of Rights, Chapter 2, South African Constitution [31]	1996	Defines human rights, including socioeconomic entitlements, principles of dignity and equality and the need for societal redress post-apartheid. Section 11 guarantees everyone the right to life. Section 27 outlines the right to access health care services, including reproductive health care, as well as emergency medical treatment, which cannot be limited. Other sections address the social determinants of health and special populations (e.g.: housing, environment, food and children).
National Health Act 61, as amended [32]	2003; 2013	‘Provides a framework for a structured uniform health system in [South Africa] taking into account the obligations imposed by the Constitution and other laws on the national, provincial and local governments with regard to health’. In a federalist system, this Act regulates the provision of health care services for public and private sectors as well as delegations to the nine provinces. Establishes The Office of Health Standards Compliance as an independent body to ensure that both public and private health establishments in South Africa comply with the required health standards.
Medicines and Related Substances Act 101, as amended [33]	1965	Regulates the manufacturing and importation of pharmaceuticals and medical devices, including pricing of same. Establishes the South African Health Products Regulatory Authority which reports to the Minister of Health.
Patents Act 57, as amended [34]	1978	Regulates patent registration and provides for voluntary and compulsory licensing through a legal process.
Medical Schemes Act 131 [35]	1998	Consolidates previous legislation concerning the health insurance industry. Establishes the Council for Medical Schemes as a juristic person to regulate, control and coordinate certain activities of the 76 different not-for-profit medical schemes, to protect the interests of members of medical schemes. It also outlines prescribed minimum benefits for 271 specific conditions, including ‘treatable’ solid organ and systemic or non-solid organ cancers (such as leukaemia and lymphoma).
Labour Relations Act 66 [36]	1995	Governs labour practices and non-discrimination in the workplace; regulates the organisational rights of trade unions, including facilitating collective bargaining; regulates the right to strike; promotes employee participation in decision-making; establishes the Commission for Conciliation, Mediation and Arbitration for independent alternative dispute resolution services as well as the Labour Court and the Labour Appeal Court to decide matters arising from the Act. Establishes grounds for incapacity due to ill health or injury, to safeguard against unfair dismissal in the workplace when sick.
Environmental Management Act 107 [37]	1998	Recognises that many inhabitants of South Africa live in an environment that is harmful to their health and well-being; regulates exposure to toxic substances in the environment, including the promotion of community participation in affected areas.
National Liquor Act 59 [38]	2003	Regulates the alcohol industry and the sale of alcohol.
Tobacco Products Control Amendment Act 63 [39]	2008	Prohibits smoking in public places and regulates the production, importation, advertisement, packaging and labelling of tobacco products; prohibits the sale of tobacco products to minors
Regulation no. 380 of the National Health Act no. 61 of 2003: National Cancer Registration Regulation [40]	2011	Formally establishes the National Cancer Registry (NCR) for cancer surveillance, with a compulsory legal requirement for every health care worker, in both public and private sectors, to report confirmed cancer cases to the NCR. Aligns public health reporting of cancer to international standards; and, mandates the NCR to shift from pathology-based surveillance to establish population-based cancer registries for the country [41].
National Public Health Institute of South Africa (NAPHISA) Act 1 of 2020 [42]	This bill has been signed by the President however, it has not yet been enacted.	The Act establishes a National Public Health Institute to coordinate disease prevention, control and surveillance, which could include cancer. It also ‘provide[s] for specialised public health services, public health interventions, training and research directed towards the major health challenges affecting the population of the Republic’.
NHI Bill [B11-2019] [43]	Passed the National Assembly in June 2023, the Bill is pending concurrence in the National Council of Provinces	Parliament passed this law to extend UHC to all South Africans through the establishment of a single funding pool. Details of this new regime must still be worked out and are hotly contested.
**Cancer-specific policies**		
Cervical Cancer Prevention and Control Policy [44]	1998, updated in 2017	Introduces mass HPV vaccination through a school-based program for girls prior to the initiation of sexual activity for primary prevention of cervical cancer; introduces evidence-based secondary prevention screening in the public sector at primary care level with PAP smears/cytology augmented by HPV DNA-based screening where resources permit; provides additional screening benefits for women living with Human immunodeficiency virus/Acquired immunodeficiency syndrome (HIV/AIDS). This policy is fully integrated into primary health care (PHC) service delivery, and budgeted for by provinces. Related treatment services are referred to treatment centres at tertiary level within provinces.
Breast Cancer Prevention and Control Policy [45]	Approved in 2017	Lays out 22 objectives to promote public awareness and access to early breast cancer detection, diagnosis, appropriate treatment and palliative care services; establishes specialised ‘units’ in tertiary care centres in the public sector for timely diagnosis and treatment of breast cancer to improve survival outcomes. However, these services are not integrated into primary levels of care across the provinces and no budgets are allocated for services within primary and secondary levels of care.
